# Patient-reported outcomes of taste alterations and quality of life in patients with nasopharyngeal carcinoma

**DOI:** 10.12669/pjms.39.6.7830

**Published:** 2023

**Authors:** Jie Li, Xiaoqian Shao, Rongling Yu, Lingyun Shi, Dejing Xu, Xiaoyan Yu

**Affiliations:** 1 Jie Li, The Affiliated Cancer Hospital of Nanjing Medical University, Jiangsu Cancer Hospital, Jiangsu & Jiangsu Institute of Cancer, Research & Cancer Nursing Research Sub-Center, Nursing, Research Center of Nanjing Medical University, Nanjing 210009, Jiangsu Province, P.R. China; 2 Xiaoqian Shao, The Affiliated Cancer Hospital of Nanjing Medical University, Jiangsu Cancer Hospital, Jiangsu & Jiangsu Institute of Cancer, Research & Cancer Nursing Research Sub-Center, Nursing, Research Center of Nanjing Medical University, Nanjing 210009, Jiangsu Province, P.R. China; 3 Rongling Yu, The Affiliated Cancer Hospital of Nanjing Medical University, Jiangsu Cancer Hospital, Jiangsu & Jiangsu Institute of Cancer, Research & Cancer Nursing Research Sub-Center, Nursing, Research Center of Nanjing Medical University, Nanjing 210009, Jiangsu Province, P.R. China; 4 Lingyun Shi, The Affiliated Cancer Hospital of Nanjing Medical University, Jiangsu Cancer Hospital, Jiangsu & Jiangsu Institute of Cancer, Research & Cancer Nursing Research Sub-Center, Nursing, Research Center of Nanjing Medical University, Nanjing 210009, Jiangsu Province, P.R. China; 5 Dejing Xu, The Affiliated Cancer Hospital of Nanjing Medical University, Jiangsu Cancer Hospital, Jiangsu & Jiangsu Institute of Cancer, Research & Cancer Nursing Research Sub-Center, Nursing, Research Center of Nanjing Medical University, Nanjing 210009, Jiangsu Province, P.R. China; 6 Xiaoyan Yu, The Affiliated Cancer Hospital of Nanjing Medical University, Jiangsu Cancer Hospital, Jiangsu & Jiangsu Institute of Cancer, Research & Cancer Nursing Research Sub-Center, Nursing, Research Center of Nanjing Medical University, Nanjing 210009, Jiangsu Province, P.R. China

**Keywords:** Patient-reported outcomes, Taste alterations, Quality of life, Nasopharyngeal carcinoma, CRT

## Abstract

**Objective:**

To investigate patient-reported outcomes of taste alterations and quality of life (QoL) in patients with nasopharyngeal carcinoma (NPC).

**Methods:**

In this single-center retrospective study medical records of 191 patients with NPC undergoing chemoradiotherapy (CRT) in the Department of Radiotherapy, Jiangsu Cancer Hospital, the Affiliated Cancer Hospital of Nanjing Medical University from January 2021 to December 2021 were reviewed. A total of 120 patients met eligibility criteria and were included. The taste alterations and QoL at multiple time points during radiotherapy (RT) were compared.

**Results:**

There were significant differences in the intensity of taste, discomfort, phantogeusia and parageusia or overall taste alterations at multiple time points during CRT (p-Value<0.001). These four parameters were significantly higher two or four weeks after CRT, or at the end of CRT compared to before CRT (p-Value <0.001). The intensity of taste, discomfort, phantogeusia and parageusia or overall taste alterations were all significantly higher four weeks after CRT compared to two weeks after CRT (p-Value <0.001), and at the end of CRT compared to four weeks after CRT (p-Value <0.001). The chemotherapy-induced taste alteration scale (CiTAS) scores were highest at the end of CRT (p-Value <0.001). There were significant differences in QoL at multiple time points during CRT (p-Value <0.001), and each parameter differed significantly at various time points (p <0.05). The QoL of all areas at the end of CRT were significantly higher than those before CRT, or two or four weeks after CRT (p-Value <0.001).

**Conclusions:**

In patients with NPC undergoing CRT, taste alterations increasingly worsen as treatment progresses, with poor QoL outcomes.

## INTRODUCTION

Nasopharyngeal carcinoma (NPC) is the most common malignant tumor of the nasopharynx. It is rare in most parts of the world, but has a high prevalence in China.^1^,^2^ Radiotherapy (RT) is the main treatment for NPC, and the five-years survival rate of patients with NPC is 76%.^3^ However, most NPC patients treated with RT will develop radiation-induced oral mucositis, with an estimated 30%-67% of patients developing severe mucositis.^4^,^5^ Symptoms of oral mucositis include dry mouth, sore throat, and altered taste.^6^ Taste disorder, defined as taste disturbance, loss of taste, decreased function of the tongue, abnormal or unpleasant taste not related to the food itself or a persistent bitter taste in the mouth, are all common complications of head and neck RT.^6^,^7^

Studies have shown that taste changes may negatively affect patients’ nutritional intake, body weight, and quality of life (QoL).^7^,^8^ Quality of life is a multidimensional concept that includes several aspects, such as physical health and psychological state, and is indicative of individual’s level of well-being. Protecting the sense of taste, ensuring that patients eat well, and enhancing physical health and the immune system during RT can improve treatment outcomes and rehabilitation.^9^ There are many studies on taste alterations caused by chemotherapy (CT) and self-management strategies. However, studies focusing on taste alterations in patients undergoing chemoradiotherapy (CRT) are scarce.

Patient-reported outcomes (PROs) refer to any report directly from a patient about their health status and treatment efficacy. This evaluation of patient’s own health status and function, provides a more realistic, reliable, and cost-effective response than observer assessment.^10^ This study analyzed PROs to better understand patient-reported occurrence of taste alterations and QoL changes in NPC patients undergoing CRT. Our results may provide evidence for improving the management of patients undergoing CRT.

## METHODS

For this single-center retrospective study, medical records of 191 NPC patients undergoing CRT in the Department of Radiotherapy, Jiangsu Cancer Hospital, the Affiliated Cancer Hospital of Nanjing Medical University from January 2021 to December 2021 were reviewed. All patients were screened based on the inclusion and exclusion criteria, and a total of 120 patients, 92 males and 28 females that met eligibility criteria were ultimately enrolled in the study.

### Ethical approval:

The study was approved by the ethics committee of Jiangsu Cancer Hospital (Approval No.: 2020-055; Date: December 3, 2020).

### Inclusion Criteria:

- Patients diagnosed with NPC by histopathological examination, receiving RT as their first treatment
- Age ≥ 18 years old.
- Patients receiving induction chemotherapy (ICT) combined with concurrent chemoradiotherapy (CCRT) or CCRT alone.
- Patients without history of severe mental illness and disorders of consciousness or taste.

### Exclusion Criteria

- Patients with recurrent NPC.
- Patients who had received surgery or RT.
- Patients with language or hearing disorder.
- Patients complicated with other severe malignant tumors of liver, kidney, or heart.
- Patients with medical history of serious digestive disease, nutritional metabolic disease, or endocrine disease.
- Patients with incomplete data.

### Treatment Methods:

All patients received CCTR. For RT, patients received 3D conformal and intensity-modulated radiotherapy (IMRT) once a day, five times a week for six to seven consecutive weeks. For CT, ICT combined with CCRT or CCRT alone patients were administered taxane, platinum and fluorouracil.

### Data Collection and Observational Indicators:

Patient baseline characteristics were collected, including age, gender, complications, and tumor staging. Information on taste alterations and QoL were collected pre-CRT, two weeks post-CRT, four weeks post-CRT and at the end of CRT.

### Taste alterations:

As there is no scale for evaluating taste alterations induced by RT, taste changes were assessed using the chemotherapy-induced taste alteration scale (CiTAS) (Chinese Version)^11^, which was adapted from the version developed by Kano (2013).^12^ The scale has 18 items, including three dimensions (intensity of taste, discomfort, and phantogeusia and parageusia). Each item is scored on a Likert scale of one (“taste normally” or “no”) to five (“unable to taste at all” or “very”), and the mean score of each dimension is calculated. A higher score is indicative of stronger symptom manifestation.

### Quality of Life:

QoL was assessed using the Chinese Version of European Organization for Research and Treatment of Cancer (EORTC) head and neck cancer module (QLQ-H&N35) (Version 1.0).^13^ The EORTC QLQ-H&N35 scale is composed of 35 items (numbered from HN1 to HN35), including seven multi-item scales (pain, swallowing, sense, speech, social eating, social contact, sexuality) and 11 single-item measures (teeth, opening mouth, dry mouth, sticky saliva, coughing, feeling ill, analgesics, nutritional supplement, feeding tube, weight gain, and weight loss).^14^ HN1-30 are scored on a four-point Likert scale (`not at all’, `a little’, `quite a bit’ and `very much’), while HN31-35 have a ‘‘no/yes’’ response format. The mean scores and standard deviations of the HRQoL scales were calculated as described in.^15^ A high score indicates a high level of symptomatology and worse QoL.^15^

Both questionnaires were conducted by trained researchers. Patients completed the questionnaire by themselves after receiving guidance and advice for some special items from the researchers. If a patient was unable to independently fill in the questionnaire due to illness or visual impairment the researchers would read the questions one by one, refraining from suggestive answers. The patient dictated the answers, and the researcher filled in the questionnaire.

### Statistical Analysis:

Statistical analysis was performed using SPSS 20.0 (IBM, USA). Measurement data normally distributed were presented as mean ± standard deviation (M±SD), and comparisons of multiple time points were analyzed by repeated ANOVA. Bonferroni test was used for pairwise comparisons within one item. Qualitative data were described as frequency and percentage (n, %). p-Value less than 0.05 was considered statistically significant.

## RESULTS

A total of 120 patients with NPC were included in this study. Of them, 92 were males (76.67%) and 28 females (23.33%). The age of the patients ranged from 28 to 69 years, with an average age of 52.51±10.10 years. Tumor stage was stage-II in 10 cases (8.33%), stage-III in 70 cases (58.33%), and stage IV in 40 cases (33.33%). Baseline characteristics are described in Table-I.

**Table-I:** Baseline characteristics.

Characteristics	Number of cases (%)
***Gender***	
Male	92 (76.67)
Female	28 (23.33)
***Age (years)***	
18-39	21 (17.50)
40-59	76 (63.33)
>59	23 (19.17)
***Complications***	
Yes	52 (43.33)
No	68 (56.67)
***Tumor stage***	
II	10 (8.33)
III	70 (58.33)
IV	40 (33.33)

We then used CiTAS) (Chinese Version)^11^ that has 18 items to evaluated CRT-related changes in taste in our cohort of patients. There were significant and gradual increase in the intensity of taste, discomfort, phantogeusia and parageusia, and overall taste alterations at multiple time points during CRT (p-Value <0.001). The intensity of taste, discomfort, phantogeusia and parageusia, or overall taste alterations at two weeks and four weeks after CRT, and at the end of CRT were significantly higher than those before CRT (p-Value <0.001). Furthermore, all these four parameters of taste alterations were significantly higher at four weeks after CRT compared to two weeks after CRT (p-Value <0.001) and significantly higher at the end of CRT compared to four weeks after CRT (p-Value <0.001). The CiTAS scores were the highest at the end of CRT (p-Value <0.001) (Table-II).

**Table-II:** Taste alterations during CRT.

Parameters	Pre-CRT	Two weeks post-CRT	Four weeks post-CRT	End of CRT	p-Value
Intensity of taste	1.0±0.28	2.30±0.77^a^	3.09±0.75^a,b^	3.84±0.62^a,b,c^	<0.001
Discomfort	1.2±0.55	2.23±0.80^a^	2.93±0.88^a,b^	3.29±0.72^a,b,c^	<0.001
Phantogeusia and parageusia	1.2±0.49	2.11±0.94^a^	3.14±0.87^a,b^	3.45±0.95^a,b.c^	<0.001
Overall taste alterations	1.1±0.38	2.65±0.82^a^	3.45±1.11^a,b^	3.74±0.75^a,b,c^	<0.001

a P<0.001, compared with pre-CRT; ^b^P<0.001, compared with two weeks post-CRT; ^c^P<0.001, compared with four weeks post-CRT.

QoL of patients was then assessed using the EORTC QLQ-H&N35 scale that is composed of 35 items. CRT was associated with significant differences in all areas of QoL, such as, pain, dysphagia, sense problem, speech problems, etc., during CRT (p-Value <0.001) (Table-III). The QoL of all areas at the end of CRT were significantly higher than those before CRT, two weeks after CRT, and four weeks after CRT (p-Value <0.001). The scores of pain, dysphagia, sense and speech problem, trouble with social eating and social contact, and less sexuality gradually increased throughout treatment, and each item differed significantly at each time point (p-Value <0.001) (Table-III). The scores of the ten single-item measures also increased except for nutritional supplement and weight gain, and each item differed significantly at various time points (p-Value <0.05 for dental problems, and p-Value <0.001 for the other items) (Table-III).

**Table-III:** QoL during CRT.

Parameters	Pre-CRT	Two weeks post-CRT	Four weeks post-CRT	End of CRT	p-Value
Pain	3.52±0.81	27.97±3.21^a^	36.87±3.29^a,b^	37.04±3.40^a,b,c^	<0.001
Dysphagia	2.69±0.94	19.25±2.32^a^	31.19±3.10^a,b^	42.29±3.48^a,b,c^	<0.001
Sense problems	5.12±1.34	30.96±3.06^a^	46.67±3.71^a,b^	55.36±4.08^a,b,c^	<0.001
Speech problems	1.37±0.59	10.01±1.51^a^	13.65±2.27^a,b^	21.73±3.22^a,b,c^	<0.001
Trouble with social eating	5.09±1.11	22.93±2.36^a^	33.21±3.05^a,b^	44.45±3.70^a,b,c^	<0.001
Trouble with social contact	5.33±1.40	13.83±2.35^a^	20.57±3.02^a,b^	25.78±3.74^a,b,c^	<0.001
Less sexuality	20.68±2.59	36.67±3.51^a^	47.65±4.17^a,b^	50.05±4.77^a,b,c^	<0.001
Dental problems	1.44±0.50	1.53±0.50^d^	1.62±0.48^a,e^	1.76±0.43^a,b,c^	<0.001
Mouth opening	1.12±0.33	1.41±0.49^a^	1.63±0.48^a,b^	1.81±0.39^a,b,c^	<0.001
Dry mouth	1.35±0.48	2.18±0.61^a^	2.96±0.69^a,b^	3.21±0.73^a,b,c^	<0.001
Sticky saliva	1.32±0.47	2.39±0.58^a^	2.85±0.35^a,b^	3.31±0.56^a,b,c^	<0.001
Coughing	1.17±0.38	1.55±0.50^a^	1.72±0.49^a,b^	1.96±0.20^a,b,c^	<0.001
Feeling ill	1.42±0.49	1.82±0.38^a^	2.15±0.36^a,b^	2.49±0.50^a,b,c^	<0.001
Analgesics	1.09±0.29	1.35±0.48^a^	1.65±0.48^a,b^	1.87±0.33^a,b,c^	<0.001
Nutritional supplement	1.16±0.37	1.41±0.49^a^	1.80±0.40^a,b^	1.61±0.49^a,b,c^	<0.001
Weight loss	1.11±0.31	1.42±0.50^a^	1.71±0.45^a,b^	1.94±0.23^a,b,c^	<0.001
Weight gain	1.23±0.42	1.13±0.34^a^	1.11±0.31^a,b^	1.00±0.00^a,b,c^	<0.001

a P<0.001, compared with pre-CRT; ^b^P<0.001, compared with two weeks post-CRT; ^c^P<0.001, compared with four weeks post-CRT; ^d^P<0.05, compared with pre-CRT; ^e^P<0.05, compared with two weeks post-CRT.

## DISCUSSION

This study found that taste changes of patients with NPC progressively worsen as CRT progresses, and the QoL of patients was also affected. It is well known that a considerable number of patients with head and neck cancer undergoing RT can experience taste changes a few weeks after the onset of irradiation.^16^ We found that the taste of NPC patients changed significantly during CRT and these changes gradually worsened as the treatment progressed and were most severe at the end of CRT. Our observations are consistent with the findings by Irune et al.^17^ It is believed that the main reason for taste loss is that radiation leads to the inhibition of the gustatory cell cycle and mother cell death, which greatly reduces the supply of new cells to the taste buds. In addition, irradiation disrupts the connection between the flavor cells and the taste neurons that are extremely sensitive to radiation.^18^ Taste buds degeneration or atrophy is observed at a dose level of 20 Gray, and therefore, most of the taste bud structures are destroyed at therapeutic dose of radiation.^18^ Loss of taste acuity may occurs in almost all patients at a dose of 60 Gray.^19^ Moreover, RT can also cause oral mucositis, dry mouth, and mucus in the mouth and throat, which may affect the sense of taste to a certain extent.^20^

In addition, we found that CRT led to gradual aggravation of all items listed in the EORTC QLQ-H&N35 scale, except weight gain. All the oral- or eating-related symptoms that are experienced by RT patients may be due to radiation damage to the oral mucosa and taste buds, and decreased salivary gland secretion, resulting in loss or changes in taste perception. Loss of taste or taste alteration has been shown to have a profound effect on patients’ QoL, as they are associated with reduced appetite, altered food intake, and subsequent weight loss.^21^ Tsutsumi et al.^22^ found that decreased gene expression of the umami taste receptors T1R3 and T2R5 was observed in patients with severe stomatitis receiving CRT.

This decrease resulted in different degrees of decline in patients’ sensitivity to umami and sweetness. The loss of appetite may lead to under- or malnutrition that negatively impacts physical health and immunity of the patients, worsening their nutritional status and QoL.^23^ Furthermore, social eating was a challenge for NPC patients at the end of RT, supporting the findings by Dornan et al.^24^ The reason may be that patients may feel self-conscious, embarrassed or ashamed when eating in public, which may also be exacerbated by the lack of understanding from others.^24^ Additionally, sexual function of NPC patients was negatively affected by the treatment. We may hypothesize that decreased sexuality may be caused by prevalent depression and anxiety of the patients.^25^ Overall, QoL decreased in parallel to treatment progression, which was similar to the conclusions of Alvarez-Camacho et al.^26^

The findings of our study highlighted the significance of PROs of taste alterations and QoL in patients with NPC. These patient-reported outcomes can help healthcare providers to determine the severity of taste changes and factors, affecting QoL, and to select optimal strategies to reduce their impact. In terms of taste alterations, attention should be paid to the patients two weeks after CRT. For QoL, medical professionals should focus not only on physical symptoms, but also on the psychological state of the, and social support should be provided.

**Table:** DRAFT TABLE (REVISE BOTH TABLES II & III AS MENTIONED BELOW).

Parameters	Pre-CRT	Two weeks post-CRT	ANOVA	p-Value
Intensity of taste				
Discomfort				
Phantogeusia and parageusia				
Overall taste alterations				
Parameters	Pre-CRT	Four weeks post-CRT		
Intensity of taste				
Discomfort				
Phantogeusia and parageusia				
Overall taste alterations				
Parameters	Pre-CRT	End of CRT		
Intensity of taste				
Discomfort				
Phantogeusia and parageusia				
Overall taste alterations				

### Limitations:

It was a retrospective observational study with a small sample size, which may limit the validity of the findings. The study only investigated taste alterations from pre-treatment to post-treatment, and further follow-up on taste alterations was not explored. Since the change of taste is a dynamic process, longer post-treatment follow-up should be done to further explore the relationship between taste alterations and QoL.

## CONCLUSIONS

In patients with NPC undergoing CRT, taste alterations progressively worsen as treatment progresses, with poor QoL outcomes.

### Authors’ contributions:

**JL** and **XS:** Conceived and designed the study.

**RY, LS, DX** and **XY:** Collected the data and performed the analysis.

**JL** and **XS:** Were involved in the writing of the manuscript and are responsible for the integrity of the study.

All authors have read and approved the final manuscript.
